# Trimethylamine N-oxide in exercise physiology: a gut microbiota-derived signal linking metabolic stress, redox balance and cardiometabolic health

**DOI:** 10.1007/s00421-026-06297-4

**Published:** 2026-06-13

**Authors:** Robert A. Olek, Zsolt Radak

**Affiliations:** 1 Department of Athletics, Strength, and Conditioning, Poznan University of Physical Education, Krolowej Jadwigi 27/39, Poznan, 61-871 Poland; 2 Research Center for Molecular Exercise Science, Hungarian University of Sport Science, Budapest, Hungary; 3 Faculty of Sport Sciences, Waseda University, Tokorozawa, Japan

**Keywords:** Trimethylamine N-oxide, Exercise physiology, Training load, Recovery, Gut microbiota, Metabolic stress, Redox balance, Biomarkers

## Abstract

Trimethylamine N-oxide (TMAO) is a gut microbiota-derived metabolite traditionally associated with cardiovascular and metabolic disease risk; however, emerging evidence from exercise physiology and metabolomics suggests a more complex and context-dependent biological role. This narrative review synthesizes findings from human observational studies, exercise interventions, supplementation trials, and animal models to examine TMAO metabolism, physiological functions, and its relationship with physical activity, training adaptations, and metabolic stress. Circulating TMAO concentrations are determined primarily by dietary precursor availability, gut microbial metabolism, hepatic flavin monooxygenase activity, and renal clearance. Across human studies, TMAO levels do not consistently correlate with aerobic fitness, habitual physical activity, or training status. Exercise-only interventions rarely produce significant changes in circulating TMAO concentrations, whereas dietary manipulation and precursor supplementation exert substantially stronger effects. Experimental evidence nevertheless suggests that TMAO may exert context-dependent biological actions related to protein stabilization, mitochondrial energy metabolism, redox regulation, and tolerance to physiological stress. Acute endurance exercise, high training load, and exercise-induced muscle damage have also been associated with transient alterations in circulating or urinary TMAO levels, although findings remain limited and heterogeneous. Collectively, current evidence does not support interpreting TMAO solely as a pathogenic metabolite. Instead, circulating TMAO may reflect interactions among diet, gut microbiota activity, metabolic stress, and host physiological status. However, the usefulness of TMAO or the TMAO/TMA ratio as biomarkers of exercise adaptation, training load, or recovery remains to be established in controlled longitudinal human studies.

## Introduction

Regular physical activity induces coordinated adaptations across multiple physiological systems, including skeletal muscle metabolism, vascular function, immune regulation, and gut microbiota composition (Ashcroft et al. 2024). Therefore, increasing attention has been focused on circulating biomarkers, which may reflect training load, recovery status, and whole-body physiological adaptations (Haller et al. 2023). Among other molecules, trimethylamine N-oxide (TMAO) has attracted substantial attention. Originally investigated as a gut microbiota-derived metabolite linked to cardiovascular disease (Wang et al. 2011), TMAO has been widely interpreted as a pathogenic compound contributing to several non-communicable diseases (Hoseini-Tavassol et al. 2023). However, emerging observations from exercise physiology, metabolomics, and experimental studies suggest that this interpretation may be incomplete. In physically active populations, TMAO concentrations do not consistently track with aerobic fitness, training status, or cardiometabolic health (Steele et al. 2021). Instead, circulating levels appear strongly influenced by dietary precursor availability, microbial metabolism, and acute physiological stress (Alsulami et al. 2025). These inconsistencies raise an important question: rather than acting solely as a pathogenic metabolite, could TMAO reflect broader processes of systemic metabolic regulation? Therefore, the aim of this review is to critically synthesize current knowledge on TMAO within the framework of exercise physiology and to reinterpret this metabolite as a context-dependent signal of integrative physiological load rather than solely a cardiometabolic risk marker.

Literature searches were conducted using the PubMed/MEDLINE, Scopus, and Web of Science databases for studies published up to February 2026. The search strategy included the terms “trimethylamine N-oxide” or “TMAO” in combination with “exercise”, “training” and “physical activity”. Original experimental studies, clinical investigations, and relevant review articles published in English were considered, with particular attention given to studies examining the relationship between TMAO, exercise-induced physiological adaptations, cardiometabolic markers, and gut microbiota-related mechanisms.

### TMAO metabolism

Circulating TMAO is derived primarily from the metabolism of dietary nutrients containing trimethylamine (TMA) moieties, particularly choline, phosphatidylcholine, and L-carnitine. These compounds are metabolized by specific gut microbial taxa into TMA, which is subsequently absorbed into the portal circulation and oxidized in the liver to TMAO. This conversion is catalyzed mainly by flavin-containing monooxygenase 3 (FMO3). Once formed, TMAO circulates systemically and is eliminated through renal excretion (for review see Loo et al. 2022).

Consequently, circulating TMAO concentrations are regulated at multiple biological levels. Dietary substrate availability influences microbial TMA production, and supplementation with choline or L-carnitine can markedly elevate plasma TMAO levels (Wilcox et al. 2021; Olek et al. 2019). Moreover, red meat consumption affects TMAO levels, but the magnitude of this increase varies substantially between individuals, largely due to differences in gut microbiome composition (Li et al. 2022b). Additionally, alterations in microbial diversity are associated with aging (Kumar et al. 2016), and circulating TMAO concentrations increase with age (Ke et al. 2018). Furthermore, genetic factors and sex differences play an important role in regulating FMO3 expression and activity, thereby affecting hepatic TMAO production (Rossner et al. 2017). Lastly, kidney function represents a determinant of circulating concentrations. Reductions in renal clearance can elevate plasma TMAO levels, contributing to the strong associations frequently observed between TMAO and chronic disease states (Pan et al. 2023).

**Fig. 1:**
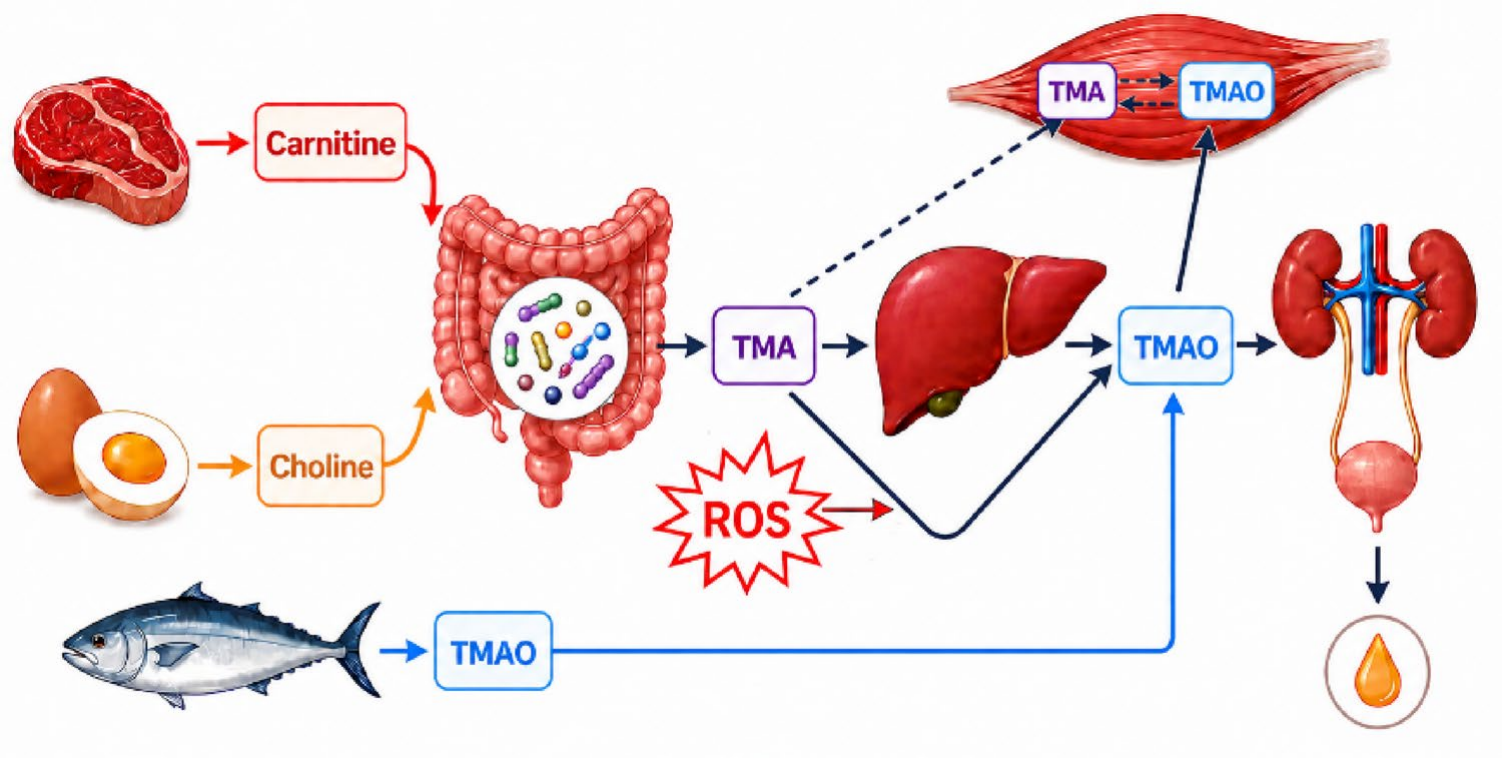
Schematic overview of the major physiological determinants of circulating TMAO concentrations, includingdiet, gut microbiota-dependent TMA formation, hepatic metabolism, renal excretion, and potential interactions withreactive oxygen species (ROS); dashed lines denote putative pathways and associations.

Together, these pathways illustrate that circulating TMAO concentrations are not determined by a single physiological process but rather emerge from a complex interaction among dietary intake, microbial metabolism, hepatic enzymatic reactivity, and renal clearance (Fig. 1).

### Biological functions of TMAO

For much of the twentieth century, TMAO was viewed merely as an end product of nitrogen metabolism destined for renal excretion (for review, see Bickel 1969). Accumulating experimental evidence indicates that it performs broader, context-dependent biological functions.

In marine organisms, TMAO acts as an important osmolyte and protein stabilizer, protecting cellular macromolecules from destabilizing environmental conditions such as high hydrostatic pressure and elevated urea concentrations (Yancey et al. 2014). Through this mechanism, TMAO promotes protein folding and structural stability, acting as a chemical chaperone that favors native protein conformations (Hamdane et al. 2016).

Experimental studies have also suggested that TMAO may participate in redox-related processes (Winter et al. 2013). Interactions between TMAO, TMA, and reactive oxygen species have been proposed to influence thiol chemistry and antioxidant pathways (Brzezinski and Zundel 1993), raising the possibility that the TMAO/TMA ratio may reflect aspects of systemic redox status.

In mammalian systems, however, the physiological significance of these mechanisms remains incompletely understood. Some experimental models suggest that chronic elevation of TMAO may contribute to cardiometabolic dysfunction, whereas others indicate potential protective roles under conditions of metabolic or oxidative stress (Naghipour et al. 2021).

### Origins of the pathogenic TMAO paradigm

The perception of TMAO changed substantially following a landmark study published in 2011 (Wang et al. 2011), which identified TMAO as a metabolite associated with atherosclerosis and cardiovascular risk. The authors demonstrated that TMAO promoted atherosclerotic lesion development in mice by enhancing cholesterol accumulation and foam cell formation. Importantly, TMAO production was absent in germ-free mice and was markedly reduced following suppression of the intestinal microbiota with broad-spectrum antibiotics. Moreover, inhibition of gut microbiota prevented the development of diet-induced atherosclerosis in mouse models.

Subsequent studies demonstrated positive associations between TMAO levels and established cardiovascular biomarkers, including N-terminal pro-B-type natriuretic peptide (NT-proBNP), high-sensitivity C-reactive protein, cardiac troponins, and markers of endothelial dysfunction, suggesting a relationship between TMAO and cardiac overload, inflammation, and vascular injury (Suzuki et al. 2016; Li et al. 2017; Chou et al. 2019).

At the same time, growing evidence suggests that interpretation of circulating TMAO should consider its precursor TMA and the TMAO/TMA ratio, which may reflect both gut microbial activity and host hepatic FMO-dependent oxidation capacity (Fennema et al. 2016; Cho and Caudill 2017). Alterations in the TMAO/TMA balance may therefore provide additional information regarding host–microbiota interactions, metabolic status, and cardiovascular risk. These findings positioned TMAO as a central metabolite within the emerging concept of the gut microbiota–organ axis (Suslov et al. 2024). Consequently, TMAO has been framed as a pathogenic mediator (Li et al. 2022a; Hoseini-Tavassol et al. 2023), prompting studies aimed at identifying strategies to reduce circulating TMAO levels (Heinrich-Sanchez and Vital 2025).

### Evidence inconsistent with the pathogenic model

#### Beneficial effects of TMAO

Despite a growing body of evidence linking elevated TMAO to adverse health outcomes, several studies suggest that TMAO may exert context-dependent beneficial effects.

Collins et al., using a mouse model closely mimicking human lipoprotein metabolism, demonstrated that higher plasma TMAO levels were inversely correlated with aortic lesion size. Moreover, TMAO did not affect macrophage cholesterol loading or cholesterol efflux, indicating no direct effect on foam cell formation (Collins et al. 2016).

In spontaneously hypertensive rats, a four- to fivefold increase in plasma TMAO concentration initiated at weaning did not exacerbate hypertension, angiopathy, or cardiac hypertrophy during early disease stages. Instead, elevated TMAO levels were associated with lower NT-proBNP concentrations, reduced left ventricular end-diastolic pressure, and attenuated cardiac fibrosis, consistent with improved diastolic function in pressure-overloaded hearts (Huc et al. 2018). Prolonged TMAO supplementation until advanced age yielded similar results, with sustained reductions in blood pressure and NT-proBNP levels and no aggravation of age-related histopathological changes, suggesting reduced cardiac workload in hypertensive rats with heart failure (Kopacz et al. 2025). Similarly, in a model of advanced heart failure, chronic TMAO administration significantly improved survival and was accompanied by favorable hemodynamic changes, including lower arterial pressure and reduced NT-proBNP levels, further supporting a cardioprotective effect associated with cardiac unloading (Gawrys-Kopczynska et al. 2020).

Mechanistically, long-term TMAO exposure may induce metabolic preconditioning-like adaptations that preserve mitochondrial function under stress conditions. In a rat model of right ventricular heart failure, chronic TMAO administration maintained mitochondrial energy metabolism by preserving fatty acid oxidation-dependent respiration and oxidative phosphorylation efficiency while promoting metabolic flexibility through a compensatory shift toward pyruvate utilization (Videja et al. 2020). Importantly, TMAO-treated animals exhibited preserved cardiac function, reduced ventricular remodeling, and lower expression of heart failure severity markers compared with untreated animals. The authors proposed that this metabolic reprogramming may improve cardiac energetic resilience during pathological stress by sustaining mitochondrial substrate utilization and preventing severe bioenergetic impairment. Furthermore, TMAO-associated cardioprotective effects have also been linked to reduced oxidative and endoplasmic reticulum stress, as well as improved vascular function (Videja et al. 2020). Consistent with these observations, a clinical study in hemodialysis patients demonstrated that TMAO elevation induced by L-carnitine supplementation was associated with reduced markers of vascular injury and oxidative stress (Fukami et al. 2015).

In addition to the reported positive associations of TMAO with cardiovascular conditions, physiologically relevant TMAO concentrations were shown to enhance blood-brain barrier integrity, protect against inflammatory insult, and improve cognitive function in mice. These effects were accompanied by reduced astrocyte and microglial reactivity (Hoyles et al. 2021).

#### The fish consumption paradox

Due to the function protecting cellular macromolecules from the destabilizing effects of high hydrostatic pressure, TMAO is naturally occurring in marine fish and other aquatic animals (Benoit and Norris 1945; Dyer 1952). Notably, fish intake elicits one of the most pronounced postprandial increases in circulating TMAO, with plasma concentrations reaching approximately 150 µmol/L within two hours of ingestion (Cho et al. 2017). This apparent contradiction raises important questions about the causal role of TMAO in cardiovascular disease (Landfald et al. 2017). If elevated TMAO were strongly pathogenic, foods that produce the largest increases in circulating TMAO would be expected to increase cardiovascular risk. Instead, a study highlighting ethnic variations revealed that Japanese patients had significantly higher circulating TMAO levels (median 9.9 µmol/L) than Caucasians (5.9 µmol/L) and South Asians (4.5 µmol/L), possibly due to their fish consumption. Despite their elevated TMAO levels, Japanese patients had the lowest 1-year mortality rate at 11%, whereas Caucasian patients had the highest mortality, at 29% (Yazaki et al. 2020).

### Human exercise physiology evidence

#### Observational studies

Regular physical activity (PA) confers substantial health benefits and reduces the risk of all-cause mortality and noncommunicable diseases (Warburton et al. 2006; Arem et al. 2015). These effects are mediated through coordinated physiological adaptations involving skeletal muscle metabolism, immune regulation, vascular function, and gut microbiota-related signaling pathways (Mallett 2025; Powers et al. 2025). Accumulating evidence indicates that PA modulates gut microbiota composition and function, promoting increased microbial diversity and altered production of bioactive metabolites involved in gut–organ communication (Clarke et al. 2014; Barton et al. 2018; Mailing et al. 2019; Torma et al. 2024; Combs et al. 2026). Consequently, healthier individuals generally report higher levels of PA, whereas patients with chronic diseases tend to exhibit higher circulating TMAO concentrations. However, despite consistent evidence supporting beneficial effects of exercise on metabolic health and gut microbiota composition, the relationship between PA and TMAO remains inconsistent and incompletely understood.

Elevated plasma TMAO levels have been reported in patients with type 2 diabetes and in individuals with ischemic stroke compared with healthy controls (Kalagi et al. 2022; Chen et al. 2022). Moreover, patients with type 2 diabetes demonstrated significantly lower PA levels than healthy individuals (Kalagi et al. 2022), and people with minor ischemic stroke engaged in significantly less PA than control subjects (Chen et al. 2022). However, in these studies, either no direct comparison between TMAO concentrations and PA levels was conducted (Chen et al. 2022) or no significant differences were detected in PA metabolic equivalent of task (MET) scores or categories across TMAO quartiles (Kalagi et al. 2022). Similarly, in a study of 250 adults aged 20–50 years, participants classified into low- and high-TMAO groups showed no significant differences in PA levels (Mirzababaei et al. 2024). Additionally, in a population-based study including 1,899 participants aged 35 years or older, no statistically significant differences in circulating TMAO concentrations were observed between hypertensive and nonhypertensive groups, despite the lower PA levels of hypertensive individuals (Guo et al. 2025).

It has been proposed that both the intensity and frequency of PA may influence TMAO concentrations (Argyridou et al. 2020; Jaaskelainen et al. 2022; Liang et al. 2025). Sedentary behavior and light PA were not associated with TMAO levels, whereas engagement in moderate to vigorous PA was associated with lower TMAO concentrations among individuals at high risk of type 2 diabetes (Argyridou et al. 2020). Similarly, in a cohort of rural northern Chinese adults aged ≥ 30 years, higher exercise intensity was associated with progressively lower TMAO levels (low: 2.4 µmol/L; moderate: 2.1 µmol/L; high: 2.0 µmol/L) (Liang et al. 2025). Consistent with these findings, Jääskeläinen et al. reported that pregnant women engaging in PA 4–7 times per week had significantly lower TMAO concentrations than women exercising ≤ 3 times per week (2.0 vs. 2.7 µmol/L, respectively) (Jaaskelainen et al. 2022). In contrast, in a cohort of 600 pregnant women (300 White Europeans and 300 South Asians; mean age of approximately 30 years), no differences in TMAO concentrations were detected between low- and high-PA categories within either ethnic group, despite European participants exhibiting both a higher prevalence of moderate-to-vigorous PA and higher circulating TMAO levels overall (Rafiq et al. 2022).

Importantly, the endurance-trained group exhibited significantly greater maximal oxygen uptake (VO₂max.) than the sedentary group (56.7 ± 8.2 vs. 39.9 ± 6.0 ml/kg/min). However, these differences in physical fitness were not associated with differences in TMAO levels. Researchers have reported no significant correlations between VO₂max. and plasma concentrations of TMAO or its dietary precursors. Furthermore, despite the endurance-trained group having a greater aerobic capacity and consuming greater amounts of TMA moieties during the high-fat diet phase, their fasting and postprandial TMAO levels remained similar to those of the sedentary group. These data indicate that high aerobic fitness alone does not necessarily predict lower or different TMAO levels in healthy young men (Steele et al. 2021).

Nevertheless, PA is frequently treated as a confounding variable in statistical analyses. Adjustment for PA aims to account for lifestyle-related differences that may influence metabolic outcomes, ensuring that the observed associations between TMAO and parameters such as abdominal obesity or triglyceride levels are not driven by variations in PA (Mirzababaei et al. 2024). Notably, even after adjustment for PA, TMAO concentrations remained significantly associated with hypertension incidence across all the applied models (Guo et al. 2025).

#### Exercise intervention studies

Intervention studies examining the effects of structured exercise training on circulating TMAO concentrations indicate that exercise performed in isolation does not elicit consistent or statistically significant changes in TMAO levels. This pattern has been observed across multiple exercise modalities, including aerobic, resistance, high-intensity interval, and multicomponent training programs (Table 1).

**Table 1:** Human exercise intervention studies and their effects on circulating TMAO levels

References	Participants	Study design	Exercise protocol	Sampling / Condition	Dietary standardization	Renal function	Overall TMAO effect	Notes / interpretation
Exercise-only interventions								
Brandao et al. (2024)	14 sedentary women with obesity (BMI 30–40 kg/m²), aged 20–40 years	single-arm pre-post intervention (without control group)	combined aerobic and resistance training; 55 min/session; 3×/week for 8 weeks	plasma / fasting	habitual diet maintained	creatinine within normal range	decreased	TMAO decrease independent of weight loss
Exercise + dietary intervention								
Erickson et al. (2019)	16 adults with obesity (mean age ~ 66 years, insulin-resistant patients)	RCT (hypocaloric diet group vs. eucaloric diet group)	treadmill or cycle ergometer; 50–60 min/session; 5×/week for 12 weeks at 80–85% HRmax,	plasma / fasting	prescribed hypocaloric or eucaloric diet	not reported (exclusion “major diseases”)	unchanged	TMAO decreased in hypocaloric (‑31%); TMAO increased in eucaloric (+ 32%)
Battillo and Malin (2023)	23 sedentary women with obesity (mean age ~ 48 years)	RCT (low-calorie diet vs. low-calorie diet + interval training)	interval cycling; 3 min at 90% and 3 min at 50% HRpeak; 12 sessions for 13 days	plasma / fasting	low-calorie diet intervention	chronic kidney disease excluded	unchanged	baseline TMAO predicts responsiveness
Exercise + supplementation studies								
Sawicka et al. (2022)	37 healthy women aged 60–75 years	RCT (three groups: L-carnitine + leucine, leucine alone, and control)	resistance training; 2×/week for 24 weeks	plasma / fasting	dietary intake monitored by 3-day food records	kidney disease excluded	supplementation-dependent	TMAO unchanged in control and placebo groups; TMAO increased in carnitine group
Olek et al. (2023)	27 postmenopausal women, untreated for osteoporosis	RCT (double-blind)	resistance training; 45–60 min/session; 2×/week for 12 weeks	plasma / fasting	dietary intake monitored by 3-day food records	kidney disease excluded	supplementation-dependent	TMAO unchanged in placebo group; TMAO increased in carnitine group
Baptista et al. (2024)	41 older adults (mean age 72.1 years) with high cardiovascular disease risk	RCT (ancillary study to a larger three-arm, double-masked clinical trial)	aerobic, resistance, flexibility, and balance; 80 min/session; 2×/week for 12 weeks	plasma / fasting	not reported	not reported (exclusion “severe medical conditions”)	increased over time	TMAO unchanged in placebo group

RCT – randomized controlled trial; HRmax – maximal heart rate; HRpeak – peak heart rate

A significant reduction in plasma TMAO concentrations (from 8.5 to 5.1 µmol/L) was reported in a noncontrolled study following an 8-week combined resistance and aerobic exercise intervention in women with obesity (Brandao et al. 2024). Notably, this reduction occurred in the absence of significant weight loss and coincided with a significant improvement in VO₂max. However, this finding was not replicated in other interventions of longer duration (12–24 weeks), which reported no significant changes in TMAO concentrations following exercise training alone (Sawicka et al. 2022; Olek et al. 2023; Baptista et al. 2024).

In contrast to exercise-only interventions, combined exercise and dietary interventions demonstrated a more pronounced influence on circulating TMAO concentrations. In insulin-resistant adults, the combination of high-intensity aerobic exercise with a hypocaloric diet resulted in a 31% reduction in plasma TMAO concentrations, whereas the same exercise protocol performed under eucaloric conditions was associated with a 32% increase. Despite these opposing directional changes, absolute TMAO concentrations did not differ significantly within or between groups (Erickson et al. 2019). Similarly, a 13-day high-intensity interval training intervention combined with a low-calorie diet in women with obesity did not result in a significant change in the mean plasma TMAO concentration (Battillo and Malin 2023).

Evidence from supplementation studies further underscores the dominant role of dietary precursors over exercise in determining circulating TMAO levels. Across trials involving postmenopausal and healthy older women, L-carnitine supplementation resulted in marked increases in plasma TMAO concentrations, irrespective of training duration (Sawicka et al. 2022; Olek et al. 2023). In contrast the exercise-only and placebo groups did not exhibit significant changes in TMAO, indicating that increased precursor availability can override any potential modulatory effects of exercise on gut microbial TMAO production.

Conversely, in a multicomponent exercise intervention incorporating aerobic, resistance, flexibility, and balance training combined with resveratrol supplementation or placebo, Baptista et al. reported an increase in circulating TMAO concentrations over time (Baptista et al. 2024). No significant treatment-by-time interaction or main effect of resveratrol was observed. Instead, a significant main effect of time emerged, with TMAO concentrations increasing independently of supplementation, suggesting that resveratrol does not meaningfully influence TMAO regulation in this context.

#### Acute exercise and endurance studies

Although regular exercise does not appear to substantially affect TMAO metabolism, available evidence suggests that TMAO responses may emerge under conditions of prolonged physiological stress, high training load, or skeletal muscle injury.

#### Intermittent exercise

In a controlled crossover study involving 11 healthy, physically active young adults, participants completed an intermittent running protocol consisting of repeated 3-min bouts at 70% heart rate reserve interspersed with walking recovery, which was compared with a time-matched seated rest condition. Following the ingestion of 700 mg of choline, circulating TMAO concentrations increased significantly over an 8-hour post-prandial period under both conditions. However, no significant differences in the serum TMAO concentration at any time point or in the total area under the concentration-time curve were detected between the exercise and resting trials. At 8 h post-ingestion, the mean TMAO concentrations were comparable across the trials. Collectively, these findings indicate that acute intermittent exercise does not modify the gut microbial conversion of choline to TMAO, nor does it provide immediate attenuation of postprandial TMAO elevations in healthy, physically active individuals (Ong et al. 2024).

#### Prolonged endurance exercise

Amateur marathon runners were stratified into two groups on the basis of prerun plasma TMAO concentrations: a low-TMAO group (< 4.0 µmol/L) and a high-TMAO group (≥ 4.0 µmol/L). Postmarathon increases in cardiac troponin I (cTnI) and endothelin-1 (ET-1) in runners with high baseline TMAO (Siekierzycka et al. 2025) were comparable to those observed in athletes with exercise-induced hypertension (EIH) (Kim et al. 2013). These findings may suggest an association between elevated TMAO levels and exercise-related cardiovascular stress responses. Furthermore, the greater intensity and running speed of a standard marathon, compared with a 100-km ultramarathon, elicit a more pronounced release of cTnI and ET-1 in individuals with EIH, likely reflecting the substantially greater acute cardiac load imposed during marathon running (Kim et al. 2013). In line with these observations, following the 100-km race no statistically significant change in average TMAO levels was observed for the whole group of 16 runners. Significant increases were noted in the three fastest finishers of the 100-km race, with increases of 1.46-, 3.92-, and 2.52-fold (Wolyniec et al. 2019). Collectively, these findings suggest that high baseline TMAO may reflect physiological conditions associated with greater release of cardiac and vascular stress markers during marathon running.

### Supportive metabolomic and biomarker evidence

#### Urinary metabolomics

Elevated urinary TMAO levels have been reported in patients with juvenile idiopathic inflammatory myopathy, a condition characterized by chronic inflammation and structural disruption of skeletal muscle and other organs (Chung et al. 2005). Similarly, urinary metabolomic profiling following eccentric exercise identified TMAO as a discriminant metabolite in male participants, suggesting that TMAO may be responsive to exercise-induced muscle stress or altered metabolic states associated with muscle damage. However, the increase in urinary TMAO concentration did not reach statistical significance and was not observed in female participants, indicating that these findings should be interpreted cautiously (Jang et al. 2018).

#### Training load markers

Additional observational evidence from urinary metabolomic profiling of professional snowboarders across different training phases demonstrated alterations in TMAO and TMA levels (Wang et al. 2015). During a 29-hour weekly training program, increasing training intensity from 80% to 90% of maximal heart rate, together with an approximately 10% increase in training volume, was associated with significantly higher urinary TMAO levels and lower TMA concentrations. Notably, when training volume was subsequently reduced by approximately 15% while maintaining the same exercise intensity, no further significant metabolic changes were observed. Collectively, these findings may be consistent with a relationship between TMAO metabolism and physiological stress or training load. However, the available evidence remains preliminary and does not support a mechanistic role for TMAO in exercise-induced muscle adaptation or damage. Further research is required to determine whether urinary TMAO levels or the TMAO/TMA ratio may serve as biomarkers of exercise stress or metabolic adaptation.

### Mechanistic and experimental evidence

#### Animal models

Animal studies have examined the effects of exercise on metabolic outcomes under diverse experimental conditions (Table 2). Considerable methodological heterogeneity was observed across studies with respect to animal strain and sex, diet composition, TMAO dose and route of administration, exercise modality, and outcome measures. Most studies have been conducted in male mice via voluntary or forced exercise protocols. Interventions included dietary manipulation (Zhang et al. 2017, 2023), exogenous TMAO administration (Zou et al. 2024a, b, 2025), and pharmacological modulation of TMAO production (Brunt et al. 2022). The outcomes were evaluated at the metabolic, functional, molecular, and microbiome levels.

**Table 2:** Animal studies examining interactions between exercise, TMAO modulation, and physiological outcomes

References	Animal model	Primary focus	Diet / model	Exercise / training	TMAO-related intervention	Biological matrix / endpoint	Direction of TMAO effect
Exercise as a protective intervention against TMAO-induced alterations							
Zhang et al. (2017)	CD1 mice ♂, 8 weeks	exercise-induced cardioprotection in obesity	Western diet	voluntary exercise, 8 weeks	TMAO administration (drinking water 120 mg/kg)	plasma TMAO, metabolic phenotype	detrimental; TMAO attenuated exercise-induced metabolic improvements
Brunt et al. (2022)	C57BL/6N mice ♂, 8 weeks	TMAO suppression in vascular dysfunction and exercise intolerance	Western diet	none	DMB-mediated TMAO inhibition (1% DMB drinking water)	plasma TMAO, frailty and exercise performance	detrimental; DMB lowered TMAO, improved exercise tolerance and grip strength without affecting muscle mass
Zhang et al. (2023)	APP/PS1 mice ♂	cognitive impairment and the protective role of exercise	normal diet	voluntary exercise, 12 weeks	TMAO administration (dietary 0.12%)	brain pathology, neuroinflammation	detrimental; TMAO induced neuroinflammation and AD-like pathology; voluntary exercise attenuated these effects
Zou et al. (2025)	C57BL/6J mice ♂, 11 weeks	TMAO-induced cardiac dysfunction	normal diet	treadmill, moderate intensity continuous training, 8 weeks	TMAO administration (intragastric 800 mg/kg)	cardiac function, metabolomics	detrimental; TMAO impaired cardiac function and altered metabolic profiles; moderate intensity continuous training attenuated these effects
TMAO modulation and exercise performance							
Zou et al. (2024b)	C57BL/6 mice ♂, 11 weeks	oxidative stress and endurance	normal diet	swimming to exhaustion	TMAO administration (intraperitoneal 800 mg/kg)	skeletal muscle oxidative stress	potentially adaptive; TMAO increased time to exhaustion; attenuation of exercise‑induced oxidative stress in skeletal muscle
Zou et al. (2024a)	C57BL/6J mice ♂, 11 weeks	exercise metabolism	normal diet	swimming to exhaustion	TMAO administration (intragastric 800 mg/kg)	endurance metabolism	potentially adaptive; TMAO increased endurance performance; modulation of glutathione and amino‑acid metabolism
Diet-induced TMAO alterations and exercise							
Almer et al. (2023)	Sprague–Dawley rats ♀, 4 months	gut microbiota and exercise	high-fat diet	running training 30 min/day, 5×/week	none	serum TMAO, gut microbiota	context-dependent; high‑fat diet reduced serum TMAO; exercise altered microbiome diversity without affecting TMAO

DMB – 3,3-dimethyl-1-butanol (inhibitor of microbial TMA lyase)

In male mouse models generated via a Western diet, alterations in circulating TMAO levels were observed alongside changes in metabolic, inflammatory, and cardiovascular parameters (Zhang et al. 2017; Brunt et al. 2022). Voluntary exercise attenuated diet-associated metabolic impairments and reduced circulating TMAO concentrations (Zhang et al. 2017). Conversely, in a female rat model high-fat diet (HFD) feeding altered the gut microbiome diversity and reduced circulating TMAO levels, whereas exercise affected the microbial composition without changing the serum TMAO concentration (Almer et al. 2023).

The inhibition of microbial TMA formation by 3,3-dimethyl-1-butanol (DMB) treatment was associated with improvements in functional outcomes related to physical performance and frailty (Brunt et al. 2022). These effects were reported in both standard chow– and Western diet–fed animals, despite minimal changes in muscle mass or baseline metabolic markers. Interestingly, AMP-activated protein kinase (AMPK) phosphorylation in skeletal muscle was greater in DMB-treated mice fed standard chow than in control mice, despite the lack of differences in plasma TMAO levels.

Disease models provide additional insight into the interaction between TMAO and exercise. In transgenic models of Alzheimer’s disease, chronic TMAO supplementation exacerbates gut microbiota dysbiosis, impairs gut–brain barrier integrity, and accelerates neuroinflammation and neuropathology. Exercise partially attenuated these detrimental effects, restoring microbial diversity, improving synaptic function, and ameliorating cognitive decline (Zhang et al. 2023). Similarly, in models of TMAO-induced myocardial injury, structured aerobic training reverses cardiac dysfunction and normalizes metabolic pathways disrupted by TMAO, including aromatic amino acid biosynthesis and central energy metabolism (Zou et al. 2025). These findings raise the possibility that exercise may offset certain TMAO-associated alterations and influence metabolic pathways disrupted during chronic TMAO exposure.

In contrast to chronic exposure, short-term TMAO administration under conditions of extreme physical stress appears to exert protective effects (Zou et al. 2024a, b). In models of exhaustive exercise, TMAO treatment prolongs the time to fatigue during swimming tests. Biochemical analyses of skeletal muscle indicated that TMAO attenuated exercise-induced reductions in taurine, glutathione, and glutathione peroxidase activity. Protein expression analyses revealed that TMAO mitigated the exercise-associated downregulation of antioxidant and stress response proteins, including Nrf2, NQO1, HO-1, and catalase (Zou et al. 2024b). Metabolomic analyses further revealed that TMAO supports energy and redox homeostasis during intense exercise by modulating amino acid metabolism and glutathione-related pathways. Importantly, these effects are observed only in exercised animals and not in sedentary controls, reinforcing the notion that TMAO acts as a conditional modulator rather than a baseline enhancer of metabolic function (Zou et al. 2024a). Nevertheless, no studies have directly compared short-term versus long-term TMAO exposure within the same experimental framework.

#### Potential redox-related roles of TMAO and TMA

During exercise, skeletal muscle undergoes profound redox perturbations driven by contraction-induced reactive oxygen species production and activation of redox-sensitive signaling pathways. These processes alter substrate utilization, metabolite exchange, and interorgan metabolic communication (Powers et al. 2024).

The ability of TMA to react with hydrogen peroxide (H₂O₂), forming TMAO, was first demonstrated in 1899 (Dunstan and Goulding 1899). Subsequent studies showed that TMA can also nonenzymatically scavenge various reactive oxygen and nitrogen species, including superoxide anion, hypochlorite, and peroxynitrite (Winter et al. 2013), supporting a potential role for TMA in redox-related processes and antioxidant defense mechanisms. Moreover, TMAO itself has been implicated in redox processes, acting as an electron or hydrogen acceptor (Ackermann et al. 1927). In particular, its interaction with sulfhydryl (–SH) groups present in proteins and low-molecular-weight thiols, such as glutathione, leading to formation of disulfide bonds or other sulfur-containing products (Brzezinski and Zundel 1993). Interestingly, evidence from isotope-tracing study demonstrated rapid distribution of circulating TMAO into skeletal muscle in humans. Six hours after administration, approximately 19% of the ingested tracer was detected in skeletal muscle, corresponding to an isotopic enrichment of 36% for TMAO and 3.3% for TMA within the skeletal muscle metabolite pool (Taesuwan et al. 2017). Within this context, circulating metabolites that readily distribute into muscle tissue may provide a rationale for exploring potential interactions between TMAO metabolism and exercise-induced physiological stress.

The reversible interconversion between TMA and TMAO, coupled with their sensitivity to oxidative conditions, raises the possibility that the TMAO/TMA ratio could reflect aspects of systemic redox status or organismal metabolic load. This concept was proposed in early clinical observations showing that the ratio was markedly lower in cancer patients than in healthy individuals (Hinsberg and Gangl-Reuss 1942). However, because the measurements were performed in urine, they may not adequately reflect dynamic redox regulation in healthy subjects. Nevertheless, these observations raise the hypothesis that the plasma TMAO/TMA ratio may be associated with redox-linked metabolic adjustments during exercise; however, this concept has not yet been systematically examined in vivo, particularly in controlled human exercise studies.

Enzymatic conversion of TMA to TMAO may also contribute to cellular redox dynamics. Under conditions of incomplete coupling, FMO catalytic cycles can leak H₂O₂ and, less frequently, superoxide, thereby constituting a potential enzymatic source of reactive oxygen species (Siddens et al. 2014). Although FMO expression in skeletal muscle appears negligible (Taesuwan et al. 2017), altered hepatic FMO activity under pathological conditions may still contribute to systemic redox-related metabolic processes.

Importantly, current evidence is largely indirect and derives primarily from biochemical observations, metabolomic associations, and nonexercise models.

### Implications for future research

Methodological sources of variability in TMAO research include numerous biological and analytical factors that substantially influence measured metabolite concentrations. One of the strongest determinants of circulating TMAO levels is short-term dietary exposure to precursor nutrients; therefore, studies that do not rigorously control pre-sampling diet or collect detailed dietary records risk conflating acute nutritional effects with underlying physiological regulation. Renal function represents another critical factor, as TMAO is primarily eliminated through kidney excretion. Variability in renal filtration capacity thus constitutes a major systemic confounder, particularly in aging populations, clinical cohorts, and studies involving dehydration or exercise-induced fluid shifts. In addition, gut microbiome composition and stability play a significant role, since interindividual differences in microbial ecology strongly influence the capacity to convert dietary precursors into TMA. Consequently, cross-sectional studies that assess circulating TMAO without parallel microbiome characterization may overlook a key determinant of metabolite production.

An additional methodological factor is the analytical technique used for metabolite quantification. Mass spectrometry-based approaches provide high specificity and sensitivity, whereas immunoassay-based methods or indirect detection techniques may introduce greater measurement error. Nevertheless, even the use of liquid chromatography–mass spectrometry can yield variable results in the measurement of TMA, which may be particularly important when calculating the TMAO/TMA ratio. Reported TMA concentrations below 1 µmol/L appear to be physiologically plausible and less likely to be affected by analytical variability (Awwad et al. 2016; Hefni et al. 2021).

## Conclusions

Current evidence indicates that TMAO is not a universally toxic metabolite. Instead, its physiological importance is determined by dietary context, microbial metabolism, exposure length, and the host’s metabolic state. Circulating TMAO concentrations are determined primarily by dietary precursor availability and gut microbiota activity, whereas aerobic fitness and training status alone do not consistently influence basal levels.

From a sport and exercise physiology perspective, TMAO may be better interpreted as a context-dependent signal reflecting the interaction among gut microbiota, hepatic metabolism, skeletal muscle stress, and systemic redox balance. The relationship between the TMAO/TMA ratio and systemic redox state indicates that TMAO may be a marker of the body’s total physiological burden. This view has potential implications for studying training stress, adaptation, and recovery dynamics in physically active persons and athletes.

Future research should further clarify the role of TMAO in exercise-induced systemic regulation, particularly the molecular mechanisms linking gut microbiota-derived metabolism with adaptive physiological responses. Standardized techniques, longitudinal designs, and sex-specific studies are required to determine TMAO’s usefulness as a biomarker for training load and physiological resilience in sports and exercise settings.

## Data Availability

This review is based entirely on publicly available data. All cited studies are referenced in the article and can be accessed via the original sources listed in the References section.

## References

[CR1] Ackermann D, Poller K, Linneweh W (1927) Über das Verhalten des Trimethylaminoxids im intermediären Stoffwechsel als biologischer Wasserstoffakzeptor, besonders gegenüber Sulfhydrylgruppen. Z für Biol 85:435–452

[CR2] Almer G, Semeraro MD, Meinitzer A, Enko D, Rodriguez Blanco G, Gallé B, Horvath A, Moissl-Eichinger C, Till H, Gruber H-J, Herrmann M (2023) Impact of long-term high dietary fat intake and regular exercise on serum TMAO and microbiome composition in female rats. Nutr Healthy Aging 8(1):157–170. 10.3233/NHA-220198

[CR3] Alsulami M, Alamri H, Barhoumi T, Munawar N, Alghanem B (2025) The effect of TMAO on aging-associated cardiovascular and metabolic pathways and emerging therapies. Mol Cell Biochem 480(11):5659–5669. 10.1007/s11010-025-05356-2 40694317 10.1007/s11010-025-05356-2 PMC12572090

[CR4] Arem H, Moore SC, Patel A, Hartge P, Berrington de Gonzalez A, Visvanathan K, Campbell PT, Freedman M, Weiderpass E, Adami HO, Linet MS, Lee IM, Matthews CE (2015) Leisure time physical activity and mortality: a detailed pooled analysis of the dose-response relationship. JAMA Intern Med 175(6):959–967. 10.1001/jamainternmed.2015.0533 25844730 10.1001/jamainternmed.2015.0533 PMC4451435

[CR5] Argyridou S, Bernieh D, Henson J, Edwardson CL, Davies MJ, Khunti K, Suzuki T, Yates T (2020) Associations between physical activity and trimethylamine N-oxide in those at risk of type 2 diabetes. BMJ Open Diabetes Res Care 8(2):e001359. 10.1136/bmjdrc-2020-001359 10.1136/bmjdrc-2020-001359 PMC7709505 33262105

[CR6] Ashcroft SP, Stocks B, Egan B, Zierath JR (2024) Exercise induces tissue-specific adaptations to enhance cardiometabolic health. Cell Metab 36(2):278–300. 10.1016/j.cmet.2023.12.008 38183980 10.1016/j.cmet.2023.12.008

[CR7] Awwad HM, Geisel J, Obeid R (2016) Determination of trimethylamine, trimethylamine N-oxide, and taurine in human plasma and urine by UHPLC-MS/MS technique. J Chromatogr B Analyt Technol Biomed Life Sci 1038:12–18. 10.1016/j.jchromb.2016.10.017 27776328 10.1016/j.jchromb.2016.10.017

[CR8] Baptista LC, Wilson L, Barnes S, Anton SD, Buford TW (2024) Effects of resveratrol on changes in trimethylamine-N-oxide and circulating cardiovascular factors following exercise training among older adults. Exp Gerontol 194:112479. 10.1016/j.exger.2024.112479 38871236 10.1016/j.exger.2024.112479

[CR9] Barton W, Penney NC, Cronin O, Garcia-Perez I, Molloy MG, Holmes E, Shanahan F, Cotter PD, O’Sullivan O (2018) The microbiome of professional athletes differs from that of more sedentary subjects in composition and particularly at the functional metabolic level. Gut 67(4):625–633. 10.1136/gutjnl-2016-313627 28360096 10.1136/gutjnl-2016-313627

[CR10] Battillo DJ, Malin SK (2023) Impact of Caloric Restriction and Exercise on Trimethylamine N-Oxide Metabolism in Women with Obesity. Nutrients 15(6):1455. 10.3390/nu15061455 10.3390/nu15061455 PMC10058428 36986183

[CR11] Benoit GJ, Norris ER (1945) Studies on Trimethylamine Oxide II. The origin of Trimethylamine Oxide in Young Salmon. J Biol Chem 158(2):439–442. 10.1016/S0021-9258(18)43149-3

[CR12] Bickel MH (1969) The pharmacology and biochemistry of N-oxides. Pharmacol Rev 21(4):325–355. 10.1016/S0031-6997(25)06876-0 4902323

[CR13] Brandao CFC, Krempf M, Giolo de Carvalho F, Aguesse A, Junqueira-Franco MVM, Batitucci G, de Freitas EC, Noronha NY, Rodrigues GDS, Junqueira GP, Borba DA, Billon-Crossouard S, Croyal M, Marchini JS (2024) Sphingolipid and Trimethylamine-N-Oxide (TMAO) Levels in Women with Obesity after Combined Physical Training. Metabolites 14(8):398. 10.3390/metabo14080398 10.3390/metabo14080398 PMC11355985 39195494

[CR14] Brunt VE, Greenberg NT, Sapinsley ZJ, Casso AG, Richey JJ, VanDongen NS, Gioscia-Ryan RA, Ziemba BP, Neilson AP, Davy KP, Seals DR (2022) Suppression of trimethylamine N-oxide with DMB mitigates vascular dysfunction, exercise intolerance, and frailty associated with a Western-style diet in mice. J Appl Physiol (1985) 133(4):798–813. 10.1152/japplphysiol.00350.2022 35952350 10.1152/japplphysiol.00350.2022 PMC9512113

[CR15] Brzezinski B, Zundel G (1993) Formation of disulphide bonds in the reaction of SH group-containing amino acids with trimethylamine N-oxide. A regulatory mechanism in proteins. FEBS Lett 333(3):331–333. 10.1016/0014-5793(93)80681-J 8224204 10.1016/0014-5793(93)80681-j

[CR16] Chen C, Qiao X, Guo J, Yang T, Wang M, Ma Y, Zhao S, Ding L, Liu H, Wang J (2022) Related factors based on non-targeted metabolomics methods in minor ischaemic stroke. Front Endocrinol (Lausanne) 13:952918. 10.3389/fendo.2022.952918 36237188 10.3389/fendo.2022.952918 PMC9552842

[CR17] Cho CE, Caudill MA (2017) Trimethylamine-N-Oxide: Friend, Foe, or Simply Caught in the Cross-Fire? Trends Endocrinol Metab 28(2):121–130. 10.1016/j.tem.2016.10.005 27825547 10.1016/j.tem.2016.10.005

[CR18] Cho CE, Taesuwan S, Malysheva OV, Bender E, Tulchinsky NF, Yan J, Sutter JL, Caudill MA (2017) Trimethylamine-N-oxide (TMAO) response to animal source foods varies among healthy young men and is influenced by their gut microbiota composition: A randomized controlled trial. Mol Nutr Food Res 61(1). 10.1002/mnfr.201600324 10.1002/mnfr.201600324 27377678

[CR19] Chou RH, Chen CY, Chen IC, Huang HL, Lu YW, Kuo CS, Chang CC, Huang PH, Chen JW, Lin SJ (2019) Trimethylamine N-Oxide, Circulating Endothelial Progenitor Cells, and Endothelial Function in Patients with Stable Angina. Sci Rep 9(1):4249. 10.1038/s41598-019-40638-y 30862856 10.1038/s41598-019-40638-y PMC6414518

[CR20] Chung YL, Rider LG, Bell JD, Summers RM, Zemel LS, Rennebohm RM, Passo MH, Hicks J, Miller FW, Scott DL, Juvenile Dermatomyositis Disease Activity Collaborative Study G (2005) Muscle metabolites, detected in urine by proton spectroscopy, correlate with disease damage in juvenile idiopathic inflammatory myopathies. Arthritis Rheum 53(4):565–570. 10.1002/art.21331 16082628 10.1002/art.21331

[CR21] Clarke SF, Murphy EF, O’Sullivan O, Lucey AJ, Humphreys M, Hogan A, Hayes P, O’Reilly M, Jeffery IB, Wood-Martin R, Kerins DM, Quigley E, Ross RP, O’Toole PW, Molloy MG, Falvey E, Shanahan F, Cotter PD (2014) Exercise and associated dietary extremes impact on gut microbial diversity. Gut 63(12):1913–1920. 10.1136/gutjnl-2013-306541 25021423 10.1136/gutjnl-2013-306541

[CR22] Collins HL, Drazul-Schrader D, Sulpizio AC, Koster PD, Williamson Y, Adelman SJ, Owen K, Sanli T, Bellamine A (2016) L-Carnitine intake and high trimethylamine N-oxide plasma levels correlate with low aortic lesions in ApoE(-/-) transgenic mice expressing CETP. Atherosclerosis 244:29–37. 10.1016/j.atherosclerosis.2015.10.108 26584136 10.1016/j.atherosclerosis.2015.10.108

[CR23] Combs D, Landeros K, Garza K, Azari H, Abdelrahman M, Albracht-Schulte K (2026) Exercise intensity as a modulator of gut microbiota and host metabolic health in obesity. Gut Microbes 18(1):2661415. 10.1080/19490976.2026.2661415 42010766 10.1080/19490976.2026.2661415 PMC13102036

[CR24] Dunstan WR, Goulding E (1899) The action of hydrogen peroxide on secondary and tertiary aliphatic amines. Formation of alkylated hydroxylamines and oxamines. J Chem Soc Trans 75:1004–1011. 10.1039/CT8997501004

[CR25] Dyer WJ (1952) Amines in Fish Muscle. VI. Trimethylamine Oxide Content of Fish and Marine Invertebrates. J Fish Res Board Can 8c(5):314–324. 10.1139/f50-020

[CR26] Erickson ML, Malin SK, Wang Z, Brown JM, Hazen SL, Kirwan JP (2019) Effects of Lifestyle Intervention on Plasma Trimethylamine N-Oxide in Obese Adults. Nutrients 11(1):179. 10.3390/nu11010179 10.3390/nu11010179 PMC6356515 30654453

[CR27] Fennema D, Phillips IR, Shephard EA (2016) Trimethylamine and Trimethylamine N-Oxide, a Flavin-Containing Monooxygenase 3 (FMO3)-Mediated Host-Microbiome Metabolic Axis Implicated in Health and Disease. Drug Metab Dispos 44(11):1839–1850. 10.1124/dmd.116.070615 27190056 10.1124/dmd.116.070615 PMC5074467

[CR28] Fukami K, Yamagishi S, Sakai K, Kaida Y, Yokoro M, Ueda S, Wada Y, Takeuchi M, Shimizu M, Yamazaki H, Okuda S (2015) Oral L-carnitine supplementation increases trimethylamine-N-oxide but reduces markers of vascular injury in hemodialysis patients. J Cardiovasc Pharmacol 65(3):289–295. 10.1097/FJC.0000000000000197 25636076 10.1097/FJC.0000000000000197

[CR29] Gawrys-Kopczynska M, Konop M, Maksymiuk K, Kraszewska K, Derzsi L, Sozanski K, Holyst R, Pilz M, Samborowska E, Dobrowolski L, Jaworska K, Mogilnicka I, Ufnal M (2020) TMAO, a seafood-derived molecule, produces diuresis and reduces mortality in heart failure rats. Elife 9:e57028. 10.7554/eLife.57028 10.7554/eLife.57028 PMC7334024 32510330

[CR30] Guo S, Bai H, Han Y, Wu Y, Peng R, Zhang X, Liang B, Zhao Q, Ma M, Zhang P, Zheng L (2025) Association of the gut microbe-dependent trimethylamine N-oxide and its precursors with risk of hypertension: a cross-sectional study in rural northeastern China. Nutr Metab Cardiovasc Dis 35(9):104032. 10.1016/j.numecd.2025.104032 40287312 10.1016/j.numecd.2025.104032

[CR31] Haller N, Behringer M, Reichel T, Wahl P, Simon P, Kruger K, Zimmer P, Stoggl T (2023) Blood-Based Biomarkers for Managing Workload in Athletes: Considerations and Recommendations for Evidence-Based Use of Established Biomarkers. Sports Med 53(7):1315–1333. 10.1007/s40279-023-01836-x 37204619 10.1007/s40279-023-01836-x PMC10197055

[CR32] Hamdane D, Velours C, Cornu D, Nicaise M, Lombard M, Fontecave M (2016) A chemical chaperone induces inhomogeneous conformational changes in flexible proteins. Phys Chem Chem Phys 18(30):20410–20421. 10.1039/c6cp03635j 27401114 10.1039/c6cp03635j

[CR33] Hefni ME, Bergstrom M, Lennqvist T, Fagerstrom C, Witthoft CM (2021) Simultaneous quantification of trimethylamine N-oxide, trimethylamine, choline, betaine, creatinine, and propionyl-, acetyl-, and L-carnitine in clinical and food samples using HILIC-LC-MS. Anal Bioanal Chem 413(21):5349–5360. 10.1007/s00216-021-03509-y 34258650 10.1007/s00216-021-03509-y PMC8405501

[CR34] Heinrich-Sanchez Y, Vital M (2025) Trimethylamine-N-oxide formation, the bacterial taxa involved and intervention strategies to reduce its concentration in the human body. Ann Med 57(1):2525403. 10.1080/07853890.2025.2525403 40598778 10.1080/07853890.2025.2525403 PMC12224733

[CR35] Hinsberg K, Gangl-Reuss E (1942) Über die Trimethylamin-und Trimethylaminoxydausscheidung im Harn von Carcinomkranken. Z für Krebsforschung 52(3):227–233. 10.1007/BF01639096

[CR36] Hoseini-Tavassol Z, Ejtahed HS, Larijani B, Hasani-Ranjbar S (2023) Trimethylamine N-Oxide as a Potential Risk Factor for Non-communicable Diseases: A Systematic Review. Endocr Metab Immune Disord Drug Targets 23(5):617–632. 10.2174/1871530323666221103120410 36330632 10.2174/1871530323666221103120410

[CR37] Hoyles L, Pontifex MG, Rodriguez-Ramiro I, Anis-Alavi MA, Jelane KS, Snelling T, Solito E, Fonseca S, Carvalho AL, Carding SR, Muller M, Glen RC, Vauzour D, McArthur S (2021) Regulation of blood-brain barrier integrity by microbiome-associated methylamines and cognition by trimethylamine N-oxide. Microbiome 9(1):235. 10.1186/s40168-021-01181-z 34836554 10.1186/s40168-021-01181-z PMC8626999

[CR38] Huc T, Drapala A, Gawrys M, Konop M, Bielinska K, Zaorska E, Samborowska E, Wyczalkowska-Tomasik A, Paczek L, Dadlez M, Ufnal M (2018) Chronic, low-dose TMAO treatment reduces diastolic dysfunction and heart fibrosis in hypertensive rats. Am J Physiol Heart Circ Physiol 315(6):H1805–H1820. 10.1152/ajpheart.00536.2018 30265149 10.1152/ajpheart.00536.2018

[CR39] Jaaskelainen T, Karkkainen O, Heinonen S, Hanhineva K, Laivuori H (2022) No association in maternal serum levels of TMAO and its precursors in pre-eclampsia and in non-complicated pregnancies. Pregnancy Hypertens 28:74–80. 10.1016/j.preghy.2022.02.008 35247822 10.1016/j.preghy.2022.02.008

[CR40] Jang HJ, Lee JD, Jeon HS, Kim AR, Kim S, Lee HS, Kim KB (2018) Metabolic Profiling of Eccentric Exercise-Induced Muscle Damage in Human Urine. Toxicol Res 34(3):199–210. 10.5487/TR.2018.34.3.199 30057694 10.5487/TR.2018.34.3.199 PMC6057290

[CR41] Kalagi NA, Thota RN, Stojanovski E, Alburikan KA, Garg ML (2022) Association between Plasma Trimethylamine N-Oxide Levels and Type 2 Diabetes: A Case Control Study. Nutrients 14(10):2093. 10.3390/nu14102093 10.3390/nu14102093 PMC9148165 35631234

[CR42] Ke Y, Li D, Zhao M, Liu C, Liu J, Zeng A, Shi X, Cheng S, Pan B, Zheng L, Hong H (2018) Gut flora-dependent metabolite Trimethylamine-N-oxide accelerates endothelial cell senescence and vascular aging through oxidative stress. Free Radic Biol Med 116:88–100. 10.1016/j.freeradbiomed.2018.01.007 29325896 10.1016/j.freeradbiomed.2018.01.007

[CR43] Kim YJ, Shin YO, Lee YH, Jee HM, Shin KA, Goh CW, Kim CH, Min YK, Yang HM, Lee JB (2013) Effects of marathon running on cardiac markers and endothelin-1 in EIH athletes. Int J Sports Med 34(9):777–782. 10.1055/s-0032-1331257 23444087 10.1055/s-0032-1331257

[CR44] Kopacz W, Jaworska K, Koper M, Ufnal M (2025) Lifelong TMAO exposure exerts hypotensive effects in aged spontaneously hypertensive rats. Front Cardiovasc Med 12:1645530. 10.3389/fcvm.2025.1645530 40951831 10.3389/fcvm.2025.1645530 PMC12425887

[CR45] Kumar M, Babaei P, Ji B, Nielsen J (2016) Human gut microbiota and healthy aging: Recent developments and future prospective. Nutr Healthy Aging 4(1):3–16. 10.3233/NHA-150002 28035338 10.3233/NHA-150002 PMC5166512

[CR46] Landfald B, Valeur J, Berstad A, Raa J (2017) Microbial trimethylamine-N-oxide as a disease marker: something fishy? Microb Ecol Health Dis 28(1):1327309. 10.1080/16512235.2017.1327309 28588431 10.1080/16512235.2017.1327309 PMC5444358

[CR49] Li XS, Obeid S, Klingenberg R, Gencer B, Mach F, Raber L, Windecker S, Rodondi N, Nanchen D, Muller O, Miranda MX, Matter CM, Wu Y, Li L, Wang Z, Alamri HS, Gogonea V, Chung YM, Tang WH, Hazen SL, Luscher TF (2017) Gut microbiota-dependent trimethylamine N-oxide in acute coronary syndromes: a prognostic marker for incident cardiovascular events beyond traditional risk factors. Eur Heart J 38(11):814–824. 10.1093/eurheartj/ehw582 28077467 10.1093/eurheartj/ehw582 PMC5837488

[CR47] Li D, Lu Y, Yuan S, Cai X, He Y, Chen J, Wu Q, He D, Fang A, Bo Y, Song P, Bogaert D, Tsilidis K, Larsson SC, Yu H, Zhu H, Theodoratou E, Zhu Y, Li X (2022a) Gut microbiota-derived metabolite trimethylamine-N-oxide and multiple health outcomes: an umbrella review and updated meta-analysis. Am J Clin Nutr 116(1):230–243. 10.1093/ajcn/nqac074 35348578 10.1093/ajcn/nqac074 PMC9257469

[CR48] Li J, Li Y, Ivey KL, Wang DD, Wilkinson JE, Franke A, Lee KH, Chan A, Huttenhower C, Hu FB, Rimm EB, Sun Q (2022b) Interplay between diet and gut microbiome, and circulating concentrations of trimethylamine N-oxide: findings from a longitudinal cohort of US men. Gut 71(4):724–733. 10.1136/gutjnl-2020-322473 33926968 10.1136/gutjnl-2020-322473 PMC8553812

[CR50] Liang J, Hao J, Qin Y, Liu O, Shao K, Zhao W, Wen J (2025) Association of Betaine, Choline, and TMAO with Type 2 Diabetes in Rural China: A Nested Case-Control Study from the Handan Eye Study (HES). Diabetes Metab Syndr Obes 18:2537–2545. 10.2147/DMSO.S522576 40740408 10.2147/DMSO.S522576 PMC12309563

[CR51] Loo RL, Chan Q, Nicholson JK, Holmes E (2022) Balancing the Equation: A Natural History of Trimethylamine and Trimethylamine-N-oxide. J Proteome Res 21(3):560–589. 10.1021/acs.jproteome.1c00851 35142516 10.1021/acs.jproteome.1c00851

[CR52] Mailing LJ, Allen JM, Buford TW, Fields CJ, Woods JA (2019) Exercise and the Gut Microbiome: A Review of the Evidence, Potential Mechanisms, and Implications for Human Health. Exerc Sport Sci Rev 47(2):75–85. 10.1249/JES.0000000000000183 30883471 10.1249/JES.0000000000000183

[CR53] Mallett G (2025) The effect of exercise and physical activity on skeletal muscle epigenetics and metabolic adaptations. Eur J Appl Physiol 125(3):611–627. 10.1007/s00421-025-05704-6 39775881 10.1007/s00421-025-05704-6

[CR54] Mirzababaei A, Mahmoodi M, Keshtkar A, Ebrahimi S, Pashayee-Khamene F, Abaj F, Radmehr M, Khalili P, Mehri Hajmir M, Mirzaei K (2024) The interaction between dietary nitrates/nitrites intake and gut microbial metabolites on metabolic syndrome: a cross-sectional study. Front Public Health 12:1398460. 10.3389/fpubh.2024.1398460 39328991 10.3389/fpubh.2024.1398460 PMC11425044

[CR55] Naghipour S, Cox AJ, Peart JN, Du Toit EF, Headrick JP (2021) Trimethylamine N-oxide: heart of the microbiota-CVD nexus? Nutr Res Rev 34(1):125–146. 10.1017/S0954422420000177 32718365 10.1017/S0954422420000177

[CR57] Olek RA, Samulak JJ, Sawicka AK, Hartmane D, Grinberga S, Pugovics O, Lysiak-Szydlowska W (2019) Increased Trimethylamine N-Oxide Is Not Associated with Oxidative Stress Markers in Healthy Aged Women. Oxid Med Cell Longev 2019:6247169. 10.1155/2019/6247169 10.1155/2019/6247169 PMC6766136 31636806

[CR56] Olek RA, Samborowska E, Wisniewski P, Wojtkiewicz P, Wochna K, Zielinski J (2023) Effect of a 3-month L-carnitine supplementation and resistance training program on circulating markers and bone mineral density in postmenopausal women: a randomized controlled trial. Nutr Metab (Lond) 20(1):32. 10.1186/s12986-023-00752-1 37533033 10.1186/s12986-023-00752-1 PMC10394783

[CR58] Ong MLY, Green CG, Rowland SN, Rider K, Sutcliffe H, Funnell MP, Salzano A, Heaney LM (2024) Effect of an acute session of intermittent exercise on trimethylamine N-oxide (TMAO) production following choline ingestion. Metabolomics 20(5):110. 10.1007/s11306-024-02177-0 39369155 10.1007/s11306-024-02177-0 PMC11455687

[CR59] Pan S, Zhao D, Duan S, Chen X (2023) The role of gut-dependent molecule trimethylamine N-oxide as a novel target for the treatment of chronic kidney disease. Int Urol Nephrol 55(7):1747–1756. 10.1007/s11255-023-03500-9 36797553 10.1007/s11255-023-03500-9

[CR61] Powers SK, Radak Z, Ji LL, Jackson M (2024) Reactive oxygen species promote endurance exercise-induced adaptations in skeletal muscles. J Sport Health Sci 13(6):780–792. 10.1016/j.jshs.2024.05.001 38719184 10.1016/j.jshs.2024.05.001 PMC11336304

[CR60] Powers SK, Goldstein E, Lategan-Potgieter R, Schrager M, Skelton M, Demirel H (2025) Health benefits of physical activity: What role does skeletal muscle-organ crosstalk play? Sports Med Health Sci 7(5):329–340. 10.1016/j.smhs.2025.02.010 40936668 10.1016/j.smhs.2025.02.010 PMC12421194

[CR62] Rafiq T, Azab SM, Anand SS, Thabane L, Shanmuganathan M, Morrison KM, Atkinson SA, Stearns JC, Teo KK, Britz-McKibbin P, de Souza RJ (2022) Sources of Variation in Food-Related Metabolites during Pregnancy. Nutrients 14(12):2503. 10.3390/nu14122503 10.3390/nu14122503 PMC9227758 35745237

[CR63] Rossner R, Kaeberlein M, Leiser SF (2017) Flavin-containing monooxygenases in aging and disease: Emerging roles for ancient enzymes. J Biol Chem 292(27):11138–11146. 10.1074/jbc.R117.779678 28515321 10.1074/jbc.R117.779678 PMC5500783

[CR64] Sawicka AK, Jaworska J, Brzeska B, Sabisz A, Samborowska E, Radkiewicz M, Szurowska E, Winklewski PJ, Szarmach A, Olek RA (2022) L-Carnitine Combined with Leucine Supplementation Does Not Improve the Effectiveness of Progressive Resistance Training in Healthy Aged Women. J Nutr Health Aging 26(10):945–953. 10.1007/s12603-022-1848-y 36259583 10.1007/s12603-022-1848-y PMC12275549

[CR65] Siddens LK, Krueger SK, Henderson MC, Williams DE (2014) Mammalian flavin-containing monooxygenase (FMO) as a source of hydrogen peroxide. Biochem Pharmacol 89(1):141–147. 10.1016/j.bcp.2014.02.006 24561181 10.1016/j.bcp.2014.02.006 PMC4116332

[CR66] Siekierzycka A, Radulska A, Wozniak M, Pelikant-Malecka I, Janaszak-Jasiecka A, Lewicka E, Kalinowski L, Olek RA (2025) Plasma cardiovascular stress biomarkers response to marathon running. Sports Med Health Sci 7(6):481–486. 10.1016/j.smhs.2024.11.005 41496807 10.1016/j.smhs.2024.11.005 PMC12766284

[CR67] Steele CN, Baugh ME, Griffin LE, Neilson AP, Davy BM, Hulver MW, Davy KP (2021) Fasting and postprandial trimethylamine N-oxide in sedentary and endurance-trained males following a short-term high-fat diet. Physiol Rep 9(16):e14970. 10.14814/phy2.14970 34405585 10.14814/phy2.14970 PMC8371342

[CR68] Suslov AV, Panas A, Sinelnikov MY, Maslennikov RV, Trishina AS, Zharikova TS, Zharova NV, Kalinin DV, Pontes-Silva A, Zharikov YO (2024) Applied physiology: gut microbiota and antimicrobial therapy. Eur J Appl Physiol 124(6):1631–1643. 10.1007/s00421-024-05496-1 38683402 10.1007/s00421-024-05496-1

[CR69] Suzuki T, Heaney LM, Bhandari SS, Jones DJ, Ng LL (2016) Trimethylamine N-oxide and prognosis in acute heart failure. Heart 102(11):841–848. 10.1136/heartjnl-2015-308826 26869641 10.1136/heartjnl-2015-308826

[CR70] Taesuwan S, Cho CE, Malysheva OV, Bender E, King JH, Yan J, Thalacker-Mercer AE, Caudill MA (2017) The metabolic fate of isotopically labeled trimethylamine-N-oxide (TMAO) in humans. J Nutr Biochem 45:77–82. 10.1016/j.jnutbio.2017.02.010 28433924 10.1016/j.jnutbio.2017.02.010

[CR71] Torma F, Kerepesi C, Jokai M, Babszki G, Koltai E, Ligeti B, Kalcsevszki R, McGreevy KM, Horvath S, Radak Z (2024) Alterations of the gut microbiome are associated with epigenetic age acceleration and physical fitness. Aging Cell 23(4):e14101. 10.1111/acel.14101 38414315 10.1111/acel.14101 PMC11019127

[CR72] Videja M, Vilskersts R, Korzh S, Cirule H, Sevostjanovs E, Dambrova M, Makrecka-Kuka M (2020) Microbiota-Derived Metabolite Trimethylamine N-Oxide Protects Mitochondrial Energy Metabolism and Cardiac Functionality in a Rat Model of Right Ventricle Heart Failure. Front Cell Dev Biol 8:622741. 10.3389/fcell.2020.622741 33520996 10.3389/fcell.2020.622741 PMC7841203

[CR74] Wang Z, Klipfell E, Bennett BJ, Koeth R, Levison BS, Dugar B, Feldstein AE, Britt EB, Fu X, Chung YM, Wu Y, Schauer P, Smith JD, Allayee H, Tang WH, DiDonato JA, Lusis AJ, Hazen SL (2011) Gut flora metabolism of phosphatidylcholine promotes cardiovascular disease. Nature 472(7341):57–63. 10.1038/nature09922 21475195 10.1038/nature09922 PMC3086762

[CR73] Wang F, Han J, He Q, Geng Z, Deng Z, Qiao D (2015) Applying (1)H NMR Spectroscopy to Detect Changes in the Urinary Metabolite Levels of Chinese Half-Pipe Snowboarders after Different Exercises. J Anal Methods Chem 2015:315217. 10.1155/2015/315217 10.1155/2015/315217 PMC4458538 26101694

[CR75] Warburton DE, Nicol CW, Bredin SS (2006) Health benefits of physical activity: the evidence. CMAJ 174(6):801–809. 10.1503/cmaj.051351 16534088 10.1503/cmaj.051351 PMC1402378

[CR76] Wilcox J, Skye SM, Graham B, Zabell A, Li XS, Li L, Shelkay S, Fu X, Neale S, O’Laughlin C, Peterson K, Hazen SL, Tang WHW (2021) Dietary Choline Supplements, but Not Eggs, Raise Fasting TMAO Levels in Participants with Normal Renal Function: A Randomized Clinical Trial. Am J Med 134(9):1160–1169. 10.1016/j.amjmed.2021.03.016 33872583 10.1016/j.amjmed.2021.03.016 PMC8410632

[CR77] Winter SE, Lopez CA, Baumler AJ (2013) The dynamics of gut-associated microbial communities during inflammation. EMBO Rep 14(4):319–327. 10.1038/embor.2013.27 23478337 10.1038/embor.2013.27 PMC3615657

[CR78] Wolyniec W, Kasprowicz K, Giebultowicz J, Korytowska N, Zorena K, Bartoszewicz M, Rita-Tkachenko P, Renke M, Ratkowski W (2019) Changes in Water Soluble Uremic Toxins and Urinary Acute Kidney Injury Biomarkers After 10- and 100-km Runs. Int J Environ Res Public Health 16(21):4153. 10.3390/ijerph16214153 10.3390/ijerph16214153 PMC6862582 31661892

[CR79] Yancey PH, Gerringer ME, Drazen JC, Rowden AA, Jamieson A (2014) Marine fish may be biochemically constrained from inhabiting the deepest ocean depths. Proc Natl Acad Sci U S A 111(12):4461–4465. 10.1073/pnas.1322003111 24591588 10.1073/pnas.1322003111 PMC3970477

[CR80] Yazaki Y, Aizawa K, Israr MZ, Negishi K, Salzano A, Saitoh Y, Kimura N, Kono K, Heaney L, Cassambai S, Bernieh D, Lai F, Imai Y, Kario K, Nagai R, Ng LL, Suzuki T (2020) Ethnic differences in association of outcomes with trimethylamine N-oxide in acute heart failure patients. ESC Heart Fail 7(5):2373–2378. 10.1002/ehf2.12777 32598563 10.1002/ehf2.12777 PMC7524106

[CR81] Zhang H, Meng J, Yu H (2017) Trimethylamine N-oxide Supplementation Abolishes the Cardioprotective Effects of Voluntary Exercise in Mice Fed a Western Diet. Front Physiol 8:944. 10.3389/fphys.2017.00944 29218015 10.3389/fphys.2017.00944 PMC5703864

[CR82] Zhang Y, Wang G, Li R, Liu R, Yu Z, Zhang Z, Wan Z (2023) Trimethylamine N-oxide aggravated cognitive impairment from APP/PS1 mice and protective roles of voluntary exercise. Neurochem Int 162:105459. 10.1016/j.neuint.2022.105459 36460238 10.1016/j.neuint.2022.105459

[CR84] Zou H, Gong L, Wang Z, Huang C, Luo Y, Jia X, Yu J, Lin D, Zhang Y (2024a) Effects of Trimethylamine N-Oxide in Improving Exercise Performance in Mice: A (1)H-NMR-Based Metabolomic Analysis Approach. Molecules 29(17):4128. 10.3390/molecules29174128 10.3390/molecules29174128 PMC11397221 39274977

[CR85] Zou H, Zhou Y, Gong L, Huang C, Liu X, Lu R, Yu J, Kong Z, Zhang Y, Lin D (2024b) Trimethylamine N-Oxide Improves Exercise Performance by Reducing Oxidative Stress through Activation of the Nrf2 Signaling Pathway. Molecules 29(4):759. 10.3390/molecules29040759 10.3390/molecules29040759 PMC10893042 38398511

[CR83] Zou H, Gong L, Huang C, Lin D, Zhang Y (2025) The Protective Mechanism of Moderate Intensity Continuous Training on TMAO-Induced Myocardial Injury Based on NMR Metabolomics. Int J Mol Sci 26(18):8902. 10.3390/ijms26188902 10.3390/ijms26188902 PMC12470193 41009469

